# Association of non-exercise physical activity in mid- and late-life with cognitive trajectories and the impact of APOE ε4 genotype status: the Mayo Clinic Study of Aging

**DOI:** 10.1007/s10433-019-00513-1

**Published:** 2019-04-12

**Authors:** Janina Krell-Roesch, Jeremy A. Syrjanen, Maria Vassilaki, Bettina Barisch-Fritz, Sandra Trautwein, Klaus Boes, Alexander Woll, Walter K. Kremers, Mary M. Machulda, Michelle M. Mielke, David S. Knopman, Ronald C. Petersen, Yonas E. Geda

**Affiliations:** 1 Translational Neuroscience and Aging Laboratory, Mayo Clinic, Scottsdale, AZ USA; 2 Institute of Sports and Sports Science, Karlsruhe Institute of Technology, Karlsruhe, Germany; 3 Department of Health Sciences Research, Mayo Clinic, Rochester, MN USA; 4 Department of Psychiatry and Psychology, Mayo Clinic, Rochester, MN USA; 5 Department of Neurology, Mayo Clinic, Rochester, MN USA; 6 Department of Psychiatry and Psychology, Mayo Clinic, Scottsdale, AZ USA; 7 Department of Neurology, Mayo Clinic, Scottsdale, AZ USA

**Keywords:** Non-exercise physical activity, Cognitive trajectories, APOE ε4, Population-based study

## Abstract

In this study derived from the population-based Mayo Clinic Study of Aging, we investigated whether non-exercise physical activity (PA) was associated with global and domain-specific cognitive trajectories (memory, language, visuospatial skills, attention) and whether the association differed by apolipoprotein E (APOE) ε4 genotype status. We included 2061 community-dwelling individuals aged ≥ 70 years (50.5% males, 26.7% APOE ε4 carriers) who were cognitively unimpaired at baseline and on whom serial cognitive data and self-reported information on non-exercise PA were available. We specifically inquired about non-exercise PA carried out at two time points, i.e., midlife (between 50 and 65 years of age) and late-life (within 1 year prior to assessment) and three intensity levels, i.e., light (e.g., laundry), moderate (e.g., scrubbing floors) and heavy (e.g., hard manual labor). Linear mixed-effect models revealed that engaging in midlife PA of moderate or heavy intensity was associated with significantly less-pronounced decline of *z*-scores in all cognitive domains. Similarly, participants that engaged in late-life moderate or heavy PA had less decline in visuospatial, attention and global *z*-scores than non-active peers. These associations varied depending on APOE ε4 carrier status, i.e., APOE ε4 non-carriers but not APOE ε4 carriers that engaged in late-life PA had less decline in cognitive *z*-scores. In contrast, engaging in midlife PA, irrespective of intensity, was significantly associated with less decline in memory function only among APOE ε4 carriers.

## Introduction

Physical activity is a modifiable lifestyle factor that has been associated with cognitive function in older age. Both cross-sectional (e.g., Iso-Markku et al. 2018; Kerr et al. 2013; Middleton et al. 2010) and longitudinal studies (e.g., Gaertner et al. 2018; Stubbs et al. 2017; Willey et al. 2016) have investigated the associations between physical activity and cognition. Overall, there appears to be evidence for an association between engagement in physical activity with more preserved cognitive function and with lower risk of cognitive impairment. In line with this, meta-analyses including randomized controlled trials have shown that engaging in physical activity might be associated with better cognition (Colcombe and Kramer 2003; Etnier et al. 1997; Kramer and Colcombe 2018; Smith et al. 2010). However, investigators have also reported conflicting results or have implied that physical activity may only be beneficial for certain cognitive domains. For example, a recent systematic review found that moderate- and vigorous-intensity physical activities were associated with executive function, memory and global cognition but not attention or working memory (Engeroff et al. 2018). Another meta-analysis from the Netherlands found that physical activity was associated with better executive function and memory in healthy adults, regardless of frequency or duration of sessions (Sanders et al. 2019). Several factors that might mediate or moderate the association between physical activity and cognitive function have been discussed in the literature. For example, it has been postulated that the association between physical activity and cognition may differ between males and females (Barha et al. 2017; Fagot et al. 2019). One potential reason may be that the presumed biological mechanisms underlying the beneficial effects of physical activity on cognition may differ by sex (Barha and Liu-Ambrose 2018). Furthermore, various genetic markers that are related to cognitive aging may also play a role in the association between physical activity and cognition (Brini et al. 2018). One such marker is the apolipoprotein E (*APOE*) ε4 allele, which is a well-known genetic risk factor for Alzheimer’s disease (Corder et al. 1993; Roses 1996; Saunders et al. 1993). A growing body of research has implicated that the association between physical activity and cognitive function may differ by *APOE* ε4 carrier status (Etnier et al. 2018; Rovio et al. 2005; Schuit et al. 2001).

To date, only a few studies have investigated the longitudinal association between physical activity and cognitive trajectories as derived from repeated neuropsychological assessments over time. For example, researchers from the English Longitudinal Study of Ageing have recently reported that physical activity was associated with less-pronounced decline in memory and executive function after 10 years of follow-up (Hamer et al. 2018). However, there are several research questions that still warrant further investigation. For example, little is known as to whether non-exercise physical activity, such as household- or work-related activity, is also related to longitudinal cognitive changes. In addition, we and others have previously shown that the timing of engaging in physical activity, for example midlife versus late-life, may play a role in the association between physical activity and cognitive impairment (Krell-Roesch et al. 2016; Tolppanen et al. 2015); this may also be true for non-exercise physical activity. Finally, as mentioned above, it is not clear whether the association between physical activity and cognitive performance differs between males and females, as well as between persons carrying and not carrying the *APOE* ε4 allele.

To address these knowledge gaps, we utilized existing data from the population-based Mayo Clinic Study of Aging. We investigated whether non-exercise physical activity carried out at two different times in life, i.e., midlife and late-life, is associated with subsequent changes in cognition among persons aged 70 years and older who were cognitively unimpaired at baseline. We focused on cognitive trajectories in four domains, namely memory, language, visuospatial skills, and attention, as well as global cognition. To examine a potential impact of *APOE* ε4 and sex on the association between non-exercise physical activity and cognitive trajectories, we also stratified the analyses by *APOE* ε4 genotype status and sex. We hypothesized that engagement in non-exercise physical activity in mid- or late-life would be associated with higher cognitive *z*-scores at baseline and less decline in cognitive *z*-scores over time. We further hypothesized that these associations would be more pronounced in *APOE* ε4 non-carriers as compared to carriers.

## Methods

### Design and sample

This study was derived from the ongoing, population-based Mayo Clinic Study of Aging (MCSA) in Olmsted County, Minnesota. The reader is referred elsewhere for a detailed description of the design and conduct of the MCSA (Roberts et al. 2008). We included cognitively unimpaired participants aged 70 years and older on whom self-reported information on non-exercise physical activity at baseline and serial cognitive data were available. Data reported here were collected between 2006 and 2018. It must be noted that participants that were enrolled in the study closer to data freezing will have shorter follow-up than participants that were enrolled earlier. Neuropsychological testing took place every 15 months on average, and all included participants had at least one follow-up visit. The institutional review boards of the Mayo Clinic and Olmsted Medical Center in Rochester, Minnesota approved the MCSA protocols. All study participants provided written informed consent.

### Clinical evaluation

Participants underwent a face-to-face evaluation including a neurological examination, a risk factors ascertainment, and neuropsychological testing. The reader is referred elsewhere for details on the face-to-face evaluation (Roberts et al. 2008). Briefly, the neurological evaluation comprised a neurological history review, administration of the Short Test of Mental Status (Kokmen et al. 1991), and a neurological examination. The risk factor assessment interview was conducted by a study coordinator and also included the Clinical Dementia Rating Scale (CDR) (Morris 1993) and Functional Activities Questionnaire (FAQ) (Pfeffer et al. 1982). Neuropsychological testing was administered by a psychometrist in order to assess performance in four cognitive domains: memory [(delayed recall trials from Auditory Verbal Learning Test (Rey 1964), Wechsler Memory Scale-Revised (Wechsler 1987) Logical Memory and Visual Reproduction subtests)]; language [(Boston Naming Test (Kaplan et al. 2001), category fluency (Lucas et al. 1998)]; visuospatial skills [(Wechsler Adult Intelligence Scale-Revised (Wechsler 1981) Picture Completion and Block Design subtests)]; and attention [Trail-Making Test Part B (Reitan 1958), Wechsler Adult Intelligent Scale-Revised (Wechsler 1981) Digit Symbol Substitution subtest]. An expert consensus panel consisting of physicians, study coordinators and neuropsychologists reviewed the results for each participant and determined whether a participant was cognitively unimpaired or had cognitive impairment. Individuals were considered cognitively unimpaired according to published normative data developed on this community (Ivnik et al. 1992a, b, c, d; Malec et al. 1992). For the current study, we only included participants who were cognitively unimpaired at baseline.

### Measurement of non-exercise physical activity (predictor variable)

Non-exercise physical activity was measured at baseline using a self-reported questionnaire. Details on the questionnaire have been reported elsewhere (Geda et al. 2010). Briefly, the questionnaire was derived from two validated instruments, i.e., the 1985 National Health Interview Survey and the Minnesota Heart Survey intensity codes (Folsom et al. 1985; Moss and Parsons 1986). The questionnaire assessed the intensity and frequency of non-exercise physical activities including when performed at work at two time periods: (1) in midlife, i.e., between the ages of 50 and 65 years; and (2) in late-life, i.e., within 1 year prior to cognitive assessment. The questionnaire distinguished between three intensity levels (i.e., light, moderate and heavy) and provided examples of activities for each level: (1) light activities such as laundry, vacuuming, making beds or dusting; (2) moderate activities such as scrubbing floors, washing windows, gardening or raking leaves; and (3) heavy activities such as carrying heavy objects, heavy digging, pushing a mower or hard manual labor. Thus, the questionnaire consisted of six items: light activity in midlife, moderate activity in midlife, heavy activity in midlife, light activity in late-life, moderate activity in late-life and heavy activity in late-life. Participants were asked to provide information about the frequency at which they carried out these activities: ≤ 1 time per month, 2–3 times per month, 1–2 times per week, 3–4 times per week, 5–6 times per week and daily. Our team has previously reported that the questionnaire has moderate to good internal consistency (Geda et al. 2010).

### Neuropsychological test scores (outcome variable)

We used continuous measures of cognitive performance that were not age normed. Longitudinal *z*-scores were calculated relative to the baseline scores by converting individual test scores to *z*-scores. We then created domain-specific *z*-scores by averaging the test-specific *z*-scores and also created a global *z*-score by averaging the domain-specific *z*-scores. The outcome of interest for the linear mixed-effect model analyses was the longitudinal change in cognitive measures of memory, language, attention, visuospatial skills and global cognition.

### Statistical analysis

All analyses were conducted separately for non-exercise physical activity carried out in midlife and late-life. We created four groups of participants depending on the level of intensity of non-exercise physical activity: (1) non-active (reference group; none); (2) only light intensity activity (light); (3) moderate plus less intensity activity (moderate); (4) heavy plus less intensity activity (heavy). Participants were considered active if they reported engaging in the respective activity at least 1–2 times per week. We first compared baseline characteristics between groups using Kruskal–Wallis (for continuous outcomes such as age; reported as mean and standard deviation, SD) and Chi square tests (for categorical outcomes such as male sex; reported as number and percentage, %). We then calculated linear mixed-effects models with random subject-specific intercepts and slopes to assess associations between non-exercise physical activity in midlife and late-life, as reported at baseline, and the longitudinal cognitive end points. All models included non-exercise physical activity level, time in years from baseline and their interaction. Associations between baseline non-exercise physical activity (independent variable) and cognitive trajectories (dependent variable) were adjusted for traditional confounders, i.e., age, sex, education, medical comorbidity [using the weighted Charlson index; (Charlson et al. 1987)], and whether or not the administration of the cognitive tests was the first time ever. We also conducted stratified analyses by sex (females; males) and *APOE* ε4 genotype status (ε4+, carriers; ε4−, non-carriers) which was determined in this study through standard methods (Hixson and Vernier 1990). The statistical analyses were done using the conventional two-tailed alpha level of 0.05 and performed with SAS 9.4 (SAS Institute, Inc., Cary, NC).

## Results

### Demographics

The sample consisted of 2061 participants (50.5% males). The mean (SD) age was 78.8 (5.3) years, mean (SD) years of education was 14.2 (2.8) and 547 (26.7%) participants were *APOE* ε4 carriers. Self-reported engagement in non-exercise physical activity was common in this population-based sample of older individuals. 47% of participants reported engaging in heavy and 36% in moderate intensity physical activity in midlife. When asked about late-life, 38% of participants reported engaging in moderate and 34% in light intensity physical activity. The percentage of males was higher in the heavy intensity (midlife: 68.7%; late-life: 75.5%) as well as non-active groups (midlife: 77.9%; late-life: 68.2%). The percentage of females was higher in the light intensity group (midlife: 76.2%; late-life: 69.1%) and for midlife moderate intensity (66%). Please refer to Table 1 for an overview of characteristics at baseline. A summary of cognitive test scores at baseline is provided in Table 2.

**Table 1:** Characteristics of participants at baseline

	Midlife					
	None (*N* = 68)	Light (*N* = 273)	Moderate (*N* = 748)	Heavy (*N* = 972)	Total (*N* = 2061)	*p*
Age (years)	80.08 (5.24)	78.82 (5.54)	78.70 (5.40)	78.72 (5.14)	78.77 (5.30)	0.18^1^
Male sex, *N* (%)	53 (77.9)	65 (23.8)	254 (34.0)	668 (68.7)	1040 (50.5)	**< 0.01**^**2**^
Education (years)	15.07 (3.08)	14.17 (2.52)	14.41 (2.62)	14.02 (2.97)	14.22 (2.80)	**< 0.01**^**1**^
APOEε4 carrier, *N* (%)	22 (32.4)	67 (24.7)^{2}^	207 (27.8)^{3}^	251 (26.0)^{5}^	547 (26.7)^{10}^	0.50^2^
Charlson index	3.47 (2.85)	3.31 (2.83)	3.45 (3.18)	3.68 (3.20)	3.54 (3.13)	0.28^1^
BMI (kg/m^2^)	28.18 (5.23)^{1}^	28.17 (5.56)^{4}^	27.69 (5.07)^{6}^	28.04 (4.69)^{7}^	27.93 (4.97)^{18}^	0.14^1^
Follow-up time (years)	5.00 (2.81)	5.71 (2.84)	5.81 (2.83)	5.95 (2.85)	5.83 (2.84)	0.05^1^
Diagnosis progression						
Remained CU, *N* (%)	37 (54.4)	205 (75.1)	559 (74.7)	734 (75.5)	1535 (74.5)	
MCI, *N* (%)	25 (36.8)	60 (22.0)	158 (21.1)	206 (21.2)	449 (21.8)	
Dementia, *N* (%)	4 (5.9)	8 (2.9)	30 (4.0)	31 (3.2)	73 (3.5)	
Residence						
House, *N* (%)	34 (50.0)	143 (52.4)	430 (57.5)	694 (71.4)	1301 (63.1)	
Apartment, *N* (%)	21 (30.9)	79 (28.9)	212 (28.3)	202 (20.8)	514 (24.9)	
Retirement comm., *N* (%)	10 (14.7)	42 (15.4)	83 (11.1)	62 (6.4)	197 (9.6)	
Nursing home, *N* (%)	1 (1.5)	0 (0.0)	3 (0.4)	1 (0.1)	5 (0.2)	
Cardiovascular risk factors						
Stroke, *N* (%)	4 (5.9)	11 (4.0)	36 (4.8)^{1}^	36 (3.7)	87 (4.2)^{1}^	0.62^2^
Hypertension, *N* (%)	53 (77.9)	214 (78.4)	587 (78.6)^{1}^	746 (76.7)	1600 (77.7)^{1}^	0.82^2^
Dyslipidemia, *N* (%)	53 (77.9)	239 (87.5)	626 (83.8)^{1}^	817 (84.1)	1735 (84.2)^{1}^	0.22^2^
Diabetes, *N* (%)	13 (19.1)	53 (19.4)	144 (19.3)^{1}^	186 (19.1)	396 (19.2)^{1}^	1.00^2^

	Late-life					
	None (*N* = 179)	Light (*N* = 709)	Moderate (*N* = 781)	Heavy (*N* = 392)	Total (*N* = 2061)	*p*
Age (years)	81.16 (5.45)	79.92 (5.55)	78.10 (4.94)	76.93 (4.51)	78.77 (5.30)	**< 0.01**^**1**^
Male sex, *N* (%)	122 (68.2)	219 (30.9)	403 (51.6)	296 (75.5)	1040 (50.5)	**< 0.01**^**2**^
Education (years)	13.86 (2.83)	14.14 (2.66)	14.44 (2.84)	14.07 (2.96)	14.22 (2.80)	**0.02**^**1**^
APOEε4 carrier, *N* (%)	53 (29.6)	168 (23.8)^{4}^	217 (27.9)^{3}^	109 (28.0)^{3}^	547 (26.7)^{10}^	0.20^2^
Charlson index	4.50 (3.40)	3.70 (3.23)	3.36 (2.98)	3.15 (3.01)	3.54 (3.13)	**< 0.01**^**1**^
BMI (kg/m^2^)	28.98 (5.51)^{5}^	28.13 (5.36)^{8}^	27.69 (4.90)^{5}^	27.58 (3.97)	27.93 (4.97)^{18}^	**0.04**^**1**^
Follow-up time (years)	5.11 (2.90)	5.66 (2.81)	6.00 (2.85)	6.14 (2.80)	5.83 (2.84)	**< 0.01**^**1**^
Diagnosis progression						
Remained CU, *N* (%)	118 (65.9)	515 (72.6)	592 (75.8)	310 (79.1)	1535 (74.5)	
MCI, *N* (%)	47 (26.3)	166 (23.4)	164 (21.0)	72 (18.4)	449 (21.8)	
Dementia, *N* (%)	12 (6.7)	27 (3.8)	24 (3.1)	10 (2.6)	73 (3.5)	
Residence						
House, *N* (%)	90 (50.3)	340 (48.0)	521 (66.7)	350 (89.3)	1301 (63.1)	
Apartment, *N* (%)	56 (31.3)	217 (30.6)	204 (26.1)	37 (9.4)	514 (24.9)	
Retirement comm., *N* (%)	24 (13.4)	125 (17.6)	45 (5.8)	3 (0.8)	197 (9.6)	
Nursing home, *N* (%)	3 (1.7)	2 (0.3)	0 (0.0)	0 (0.0)	5 (0.2)	
Cardiovascular risk factors						
Stroke, *N* (%)	16 (8.9)	34 (4.8)^{1}^	23 (2.9)	14 (3.6)	87 (4.2)^{1}^	**< 0.01**^**2**^
Hypertension, *N* (%)	151 (84.4)	578 (81.6)^{1}^	604 (77.3)	267 (68.1)	1600 (77.7)^{1}^	**< 0.01**^**2**^
Dyslipidemia, *N* (%)	150 (83.8)	609 (86.0)^{1}^	642 (82.2)	334 (85.2)	1735 (84.2)^{1}^	0.22^2^
Diabetes, *N* (%)	45 (25.1)	137 (19.4)^{1}^	149 (19.1)	65 (16.6)	396 (19.2)^{1}^	0.12^2^

Data presented are mean (standard deviation) unless otherwise noted. None = non-active; Light = only light intensity activity at least 1-2 times/week; Moderate = moderate plus less intensity activity at least 1–2 times/week; Heavy = heavy plus less intensity activity at least 1–2 times/week

BMI, body mass index; Diagnosis progression during follow-up; CU, cognitively unimpaired; MCI, mild cognitive impairment; Retirement comm., retirement community

^1^Kruskal-Wallis test; ^2^Chi Square test; ^{*N*}^number of missing data

Significant *p* values appear bold

**Table 2:** Cognitive test scores at baseline

	Midlife				
	None (*N* = 68)	Light (*N* = 273)	Moderate (*N* = 748)	Heavy (*N* = 972)	Total (*N* = 2061)
Memory					
AVLT delayed recall	6.37 (3.08)^{1}^	8.31 (3.34)^{4}^	7.70 (3.41)^{16}^	6.95 (3.12)^{25}^	7.39 (3.30)^{46}^
Logical memory	15.85 (6.93)	18.75 (7.11)^{6}^	17.98 (7.41)^{10}^	17.16 (7.11)^{17}^	17.62 (7.24)^{33}^
Visual reproduction	20.50 (8.33)	21.10 (7.99)^{10}^	21.38 (8.29)^{30}^	21.46 (8.24)^{32}^	21.35 (8.23)^{72}^
Language					
BNT	54.14 (4.64)^{3}^	54.42 (4.40)^{8}^	54.51 (4.51)^{32}^	54.75 (4.39)^{24}^	54.60 (4.44)^{67}^
Category fluency	38.54 (7.96)^{5}^	43.89 (9.94)^{3}^	44.54 (9.52)^{12}^	42.00 (9.09)^{12}^	43.07 (9.44)^{32}^
Attention					
WAISR-digit symbol	39.24 (8.91)^{1}^	43.88 (11.11)^{9}^	44.20 (10.31)^{41}^	41.71 (10.0)^{36}^	42.81 (10.30)^{87}^
Trail making B (reverse)	177.97 (55.08)^{1}^	196.01 (51.72)^{11}^	197.28 (45.52)^{40}^	196.34 (44.12)^{34}^	196.01 (46.19)^{86}^
Visuospatial					
WAISR-picture completion	13.31 (2.63)^{1}^	12.78 (3.34)^{9}^	12.94 (3.28)^{38}^	13.25 (3.23)^{27}^	13.08 (3.24)^{75}^
WAISR-block design	21.72 (7.55)^{1}^	22.46 (8.17)^{13}^	23.20 (8.06)^{37}^	23.69 (8.49)^{34}^	23.29 (8.27)^{85}^
*Z*-scores					
Memory	− 0.29 (0.90)	0.18 (0.98)^{4}^	0.07 (1.04)^{10}^	− 0.08 (0.97)^{16}^	0.00 (1.00)^{30}^
Language	− 0.35 (0.96)^{5}^	0.03 (1.05)^{8}^	0.08 (1.02)^{33}^	− 0.05 (0.96)^{27}^	0.00 (1.00)^{73}^
Attention	− 0.42 (1.05)^{1}^	0.06 (1.10)^{11}^	0.09 (0.99)^{43}^	− 0.06 (0.96)^{39}^	0.00 (1.00)^{94}^
Visuospatial	− 0.05 (0.88)^{2}^	− 0.12 (0.98)^{14}^	− 0.03 (0.99)^{43}^	0.06 (1.02)^{40}^	0.00 (1.00)^{99}^
Global	− 0.37 (0.97)^{7}^	0.06 (1.04)^{18}^	0.06 (1.01)^{56}^	− 0.04 (0.98)^{59}^	0.00 (1.00)^{140}^

	Late-life				
	None (*N* = 179)	Light (*N* = 709)	Moderate (*N* = 781)	Heavy (*N* = 392)	Total (*N* = 2061)
Memory					
AVLT delayed recall	7.13 (3.30)^{6}^	7.71 (3.25)^{16}^	7.39 (3.39)^{16}^	6.93 (3.13)^{8}^	7.39 (3.30)^{46}^
Logical memory	17.94 (7.30)^{5}^	17.73 (7.04)^{9}^	17.70 (7.47)^{13}^	17.13 (7.13)^{6}^	17.62 (7.24)^{33}^
Visual reproduction	19.85 (8.45)^{10}^	20.79 (7.89)^{37}^	21.95 (8.51)^{18}^	21.81 (7.99)^{7}^	21.35 (8.23)^{72}^
Language					
BNT	53.54 (5.26)^{11}^	54.40 (4.31)^{29}^	54.75 (4.48)^{21}^	55.11 (4.13)^{6}^	54.60 (4.44)^{67}^
Category fluency	39.99 (8.22)^{6}^	43.81 (9.52)^{11}^	43.59 (9.59)^{12}^	42.06 (9.15)^{3}^	43.07 (9.44)^{32}^
Attention					
WAISR-digit symbol	38.08 (8.98)^{13}^	42.68 (10.31)^{44}^	43.82 (10.46)^{22}^	43.09 (9.98)^{8}^	42.81 (10.30)^{87}^
Trail making B (reverse)	182.64 (52.77)^{12}^	192.50 (49.58)^{44}^	199.16 (43.82)^{23}^	201.66 (39.70)^{7}^	196.01 (46.19)^{86}^
Visuospatial					
WAISR-picture completion	12.81 (3.32)^{9}^	12.90 (3.27)^{39}^	13.15 (3.30)^{21}^	13.39 (3.02)^{6}^	13.08 (3.24)^{75}^
WAISR-block design	21.25 (7.67)^{10}^	22.64 (7.97)^{44}^	23.63 (8.35)^{24}^	24.62 (8.62)^{7}^	23.29 (8.27)^{85}^
*Z*-scores					
Memory	− 0.09 (1.01)^{5}^	0.02 (0.98)^{9}^	0.03 (1.05)^{10}^	− 0.07 (0.94)^{6}^	0.00 (1.00)^{30}^
Language	− 0.35 (1.02)^{11}^	0.02 (1.00)^{32}^	0.05 (1.01)^{24}^	0.01 (0.94)^{6}^	0.00 (1.00)^{73}^
Attention	− 0.42 (0.99)^{14}^	− 0.05 (1.04)^{48}^	0.10 (0.99)^{23}^	0.08 (0.91)^{9}^	0.00 (1.00)^{94}^
Visuospatial	− 0.19 (0.97)^{13}^	− 0.08 (0.98)^{51}^	0.04 (1.01)^{26}^	0.15 (1.02)^{9}^	0.00 (1.00)^{99}^
Global	− 0.33 (0.97)^{23}^	− 0.03 (1.00)^{63}^	0.07 (1.02)^{39}^	0.06 (0.94)^{15}^	0.00 (1.00)^{140}^

Data presented are mean (standard deviation) unless otherwise noted. None = non-active; Light = only light intensity activity at least 1–2 times/week; Moderate = moderate plus less intensity activity at least 1–2 times/week; Heavy = heavy plus less intensity activity at least 1–2 times/week

AVLT, Auditory Verbal Learning Test; Logical memory, Wechsler Memory Scale-Revised logical memory subtest; Visual reproduction, Wechsler Memory Scale-Revised visual reproduction subtest; BNT, Boston naming test; WAISR-Digit symbol, Wechsler Adult Intelligent Scale-Revised digit symbol substitution subtest; Trail Making B (reverse), Trail-Making Test Part B reversed scores; WAISR-Picture completion, Wechsler Adult Intelligence Scale-Revised picture completion subtest; WAISR-Block design, Wechsler Adult Intelligence Scale-Revised block design subtest

^{*N*}^Number of missing data

### Association between non-exercise physical activity and longitudinal cognitive changes

Table 3 shows estimates and standard errors of the mixed-effect models. As expected, scores for all cognitive domains decreased significantly over time, as indicated by negative coefficients for time. Participants that engaged in midlife activity at moderate and heavy intensity levels had significantly less decline than non-active participants in all cognitive domains. On average, the coefficients are slightly higher for heavy as compared to moderate intensity suggesting a stronger effect for heavy intensity. The interaction between time and light intensity was significant for language, visuospatial skills, and global cognition also indicating less-pronounced decline among participants that engage in light intensity activity in midlife as compared to non-active participants. Participants that engaged in heavy intensity activity in late-life had less decline than non-active participants in scores across all cognitive domains. Similarly, the time x late-life activity interactions were significant for moderate intensity activity with regard to visuospatial skills, attention and global cognition indicating less-pronounced decrease of scores over time for active versus non-active participants. Among participants that engaged in late-life light intensity activity, only attention *z*-scores decreased less over time than those of non-active participants.

**Table 3:** Association between non-exercise physical activity in mid- and late-life with longitudinal cognitive changes

	Memory	Language	Visuospatial	Attention	Global
Midlife					
*T*	− 0.110 (0.022)**	− 0.140 (0.021)**	− 0.077 (0.015)**	− 0.178 (0.022)**	− 0.173 (0.021)**
Light	0.273 (0.128)*	0.238 (0.124)	0.124 (0.121)	0.263 (0.121)*	0.285 (0.122)*
Moderate	0.211 (0.119)	0.283 (0.115)*	0.150 (0.112)	0.332 (0.113)**	0.316 (0.114)**
Heavy	0.232 (0.117)*	0.283 (0.113)*	0.182 (0.110)	0.353 (0.110)**	0.349 (0.111)**
*T* × light	0.045 (0.024)	0.046 (0.023)*	0.043 (0.017)**	0.030 (0.024)	0.057 (0.023)*
*T* × moderate	0.054 (0.023)*	0.059 (0.022)**	0.040 (0.016)*	0.053 (0.023)*	0.070 (0.021)**
*T* × heavy	0.053 (0.022)*	0.065 (0.021)**	0.045 (0.016)**	0.059 (0.022)**	0.076 (0.021)**
Late-life					
*T*	− 0.087 (0.014)**	− 0.096 (0.013)**	− 0.056 (0.010)**	− 0.177 (0.014)**	− 0.146 (0.013)**
Light	− 0.035 (0.080)	0.183 (0.078)*	0.173 (0.077)*	0.158 (0.077)*	0.131 (0.078)
Moderate	− 0.009 (0.079)	0.159 (0.076)*	0.139 (0.075)	0.257 (0.076)**	0.169 (0.076)*
Heavy	− 0.053 (0.086)	0.120 (0.083)	0.129 (0.082)	0.288 (0.082)**	0.162 (0.083)
*T* × light	0.022 (0.015)	0.002 (0.014)	0.013 (0.011)	0.032 (0.015)*	0.026 (0.015)
*T* × moderate	0.027 (0.015)	0.018 (0.014)	0.027 (0.010)*	0.063 (0.015)**	0.051 (0.014)**
*T* × heavy	0.043 (0.016)**	0.031 (0.015)*	0.027 (0.011)*	0.065 (0.016)**	0.061 (0.015)**

Linear mixed-effects models adjusted for age, sex, education, medical comorbidity and whether or not the administration of the cognitive tests was the first time ever; values presented are estimate (standard error)

*T*, time; Light/Moderate/Heavy, light/moderate/heavy intensity activity; ×, indicates interaction

*p < 0.05; **p < 0.01

### Stratification by APOE ε4 carrier status

Table 4 summarizes estimates and standard errors of the mixed-effect models on the association between non-exercise physical activity and longitudinal cognitive changes as stratified by *APOE* ε4 status. Engaging in midlife non-exercise physical activity of any intensity, and as opposed to being non-active, was associated with less decline in memory function among *APOE* ε4 carriers but not *APOE* ε4 non-carriers. Engagement in light and heavy intensity activity in midlife among *APOE* ε4 carriers and moderate and heavy intensity activity in midlife among *APOE* ε4 non-carriers was associated with less-pronounced decline in global cognition. There was no significant time x late-life activity interaction for *APOE* ε4 carriers on any cognitive measure. Whereas for *APOE* ε4 non-carriers, engaging in heavy intensity activity in late-life was associated with less-pronounced decline in all cognitive domains, engaging in moderate intensity activity in late was associated with less-pronounced decline in all domains except language, and engaging in light intensity activity was associated with less-pronounced decline in memory, attention and global cognition. The numeric estimates were, on average, highest for heavy intensity and lowest for light intensity.

**Table 4:** Association between non-exercise physical activity in mid- and late-life with longitudinal cognitive changes: stratified by APOE ε4 carrier status

	Memory	Language	Visuospatial	Attention	Global
Midlife					
ε4+					
*T*	− 0.182 (0.041)**	− 0.168 (0.041)**	− 0.094 (0.030)**	− 0.220 (0.044)**	− 0.219 (0.039)**
Light	0.381 (0.238)	0.405 (0.229)	0.175 (0.225)	0.409 (0.225)	0.443 (0.223)*
Moderate	0.225 (0.214)	0.465 (0.207)*	0.303 (0.202)	0.468 (0.203)*	0.500 (0.201)*
Heavy	0.213 (0.210)	0.352 (0.202)	0.315 (0.198)	0.500 (0.198)*	0.481 (0.196)*
*T* × light	0.100 (0.047)*	0.061 (0.047)	0.065 (0.034)	0.070 (0.050)	0.095 (0.044)*
*T* × moderate	0.086 (0.043)*	0.041 (0.043)	0.048 (0.031)	0.054 (0.046)	0.072 (0.041)
*T* × heavy	0.101 (0.043)*	0.073 (0.043)	0.047 (0.031)	0.066 (0.045)	0.092 (0.040)*
ε4−					
*T*	− 0.076 (0.026)**	− 0.124 (0.024)**	− 0.067 (0.018)**	− 0.155 (0.025)**	− 0.147 (0.025)**
Light	0.211 (0.153)	0.163 (0.148)	0.083 (0.144)	0.197 (0.145)	0.206 (0.147)
Moderate	0.189 (0.144)	0.208 (0.140)	0.087 (0.135)	0.271 (0.136)*	0.235 (0.138)
Heavy	0.223 (0.141)	0.239 (0.137)	0.130 (0.132)	0.290 (0.133)*	0.283 (0.135)*
*T* × light	0.016 (0.028)	0.034 (0.026)	0.032 (0.019)	0.008 (0.027)	0.034 (0.027)
*T* × moderate	0.034 (0.026)	0.058 (0.025)*	0.033 (0.018)	0.044 (0.026)	0.060 (0.025)*
*T* × heavy	0.026 (0.026)	0.055 (0.025)*	0.040 (0.018)*	0.047 (0.026)	0.059 (0.025)*
Late-life					
ε4+					
*T*	− 0.076 (0.028)**	− 0.090 (0.028)**	− 0.069 (0.021)**	− 0.197 (0.030)**	− 0.137 (0.027)**
Light	− 0.090 (0.151)	0.124 (0.146)	0.087 (0.144)	0.096 (0.144)	0.063 (0.143)
Moderate	− 0.151 (0.147)	0.123 (0.142)	0.035 (0.140)	0.094 (0.141)	0.062 (0.140)
Heavy	− 0.142 (0.162)	− 0.005 (0.157)	0.023 (0.154)	0.184 (0.155)	0.051 (0.153)
*T* × light	− 0.045 (0.031)	− 0.056 (0.031)	0.012 (0.023)	0.004 (0.034)	− 0.038 (0.030)
*T* × moderate	0.004 (0.030)	− 0.006 (0.030)	0.031 (0.022)	0.060 (0.032)	0.019 (0.029)
*T* × heavy	− 0.015 (0.033)	− 0.007 (0.033)	0.027 (0.024)	0.050 (0.035)	0.014 (0.031)
ε4−					
*T*	− 0.089 (0.016)**	− 0.097 (0.015)**	− 0.050 (0.011)**	− 0.167 (0.016)**	− 0.147 (0.015)**
Light	− 0.036 (0.095)	0.194 (0.092)*	0.209 (0.091)*	0.182 (0.092)*	0.145 (0.093)
Moderate	0.036 (0.093)	0.172 (0.091)	0.191 (0.089)*	0.326 (0.090)**	0.211 (0.091)*
Heavy	− 0.023 (0.101)	0.174 (0.098)	0.188 (0.096)	0.347 (0.097)**	0.216 (0.098)*
*T* × light	0.042 (0.017)*	0.019 (0.016)	0.011 (0.012)	0.037 (0.017)*	0.045 (0.016)**
*T* × moderate	0.035 (0.017)*	0.026 (0.016)	0.024 (0.012)*	0.062 (0.017)**	0.061 (0.016)**
*T* × heavy	0.061 (0.018)**	0.042 (0.017)*	0.025 (0.012)*	0.066 (0.018)**	0.075 (0.017)**

Linear mixed-effects models adjusted for age, sex, education, medical comorbidity and whether or not the administration of the cognitive tests was the first time ever; values presented are estimate (standard error); ε4+ = APOE ε4 carrier; ε4− = APOE ε4 non-carrier

*T*, time; Light/moderate/heavy, light/moderate/heavy intensity activity; ×, indicates interaction

*p < 0.05; **p < 0.01

### Stratification by sex

Table 5 shows estimates and standard errors of the mixed-effect models on the association between non-exercise physical activity and longitudinal cognitive changes as stratified by sex. Females that engaged in moderate and heavy intensity activity in midlife had less decline in memory function, and those that engaged in either light, moderate or heavy intensity activity in midlife had less decline in global cognition than non-active females. For males, engaging in moderate intensity activity in midlife as compared to being non-active was associated with less decline in all cognitive domains, engaging in light intensity was associated with less decline in language, visuospatial and global domains, and engaging in heavy intensity was associated with less decline in language, visuospatial, attention and global domains. For late-life activity, engaging in any intensity activity was associated with less-pronounced decline of memory, visuospatial, attention and global cognitive function among females. Whereas for males, only engaging in moderate or heavy intensity late-life activity, as compared to being non-active, was associated with less decline in attention and global cognition.

**Table 5:** Association between non-exercise physical activity in mid- and late-life with longitudinal cognitive changes: stratified by sex

	Memory	Language	Visuospatial	Attention	Global
Midlife					
*F*					
*T*	− 0.174 (0.051)**	− 0.175 (0.050)**	− 0.099 (0.035)**	− 0.186 (0.048)**	− 0.222 (0.046)**
Light	0.374 (0.257)	0.287 (0.247)	0.148 (0.241)	0.533 (0.248)*	0.416 (0.246)
Moderate	0.309 (0.252)	0.221 (0.242)	0.156 (0.236)	0.528 (0.243)*	0.374 (0.241)
Heavy	0.324 (0.254)	0.329 (0.245)	0.204 (0.238)	0.547 (0.245)*	0.437 (0.243)
*T* × light	0.097 (0.052)	0.076 (0.051)	0.061 (0.036)	0.027 (0.049)	0.094 (0.047)*
*T* × moderate	0.103 (0.051)*	0.082 (0.050)	0.059 (0.035)	0.055 (0.049)	0.108 (0.046)*
*T* × heavy	0.103 (0.052)*	0.069 (0.051)	0.061 (0.035)	0.043 (0.049)	0.103 (0.047)*
*M*					
*T*	− 0.086 (0.022)**	− 0.125 (0.019)**	− 0.071 (0.016)**	− 0.175 (0.024)**	− 0.154 (0.021)**
Light	0.218 (0.166)	0.066 (0.157)	0.061 (0.158)	0.044 (0.154)	0.135 (0.155)
Moderate	0.181 (0.136)	0.412 (0.129)**	0.148 (0.128)	0.323 (0.126)*	0.363 (0.127)**
Heavy	0.201 (0.129)	0.286 (0.122)*	0.171 (0.121)	0.332 (0.119)**	0.343 (0.120)**
*T* × light	0.039 (0.029)	0.050 (0.025)*	0.052 (0.021)*	0.060 (0.032)	0.069 (0.028)*
*T* × moderate	0.050 (0.024)*	0.063 (0.021)**	0.039 (0.018)*	0.059 (0.026)*	0.068 (0.023)**
*T* × heavy	0.038 (0.023)	0.066 (0.020)**	0.042 (0.017)*	0.068 (0.025)**	0.069 (0.022)**
Late-life					
*F*					
*T*	− 0.137 (0.028)**	− 0.128 (0.029)**	− 0.096 (0.020)**	− 0.269 (0.028)**	− 0.207 (0.027)**
Light	0.024 (0.137)	0.362 (0.134)**	0.244 (0.132)	0.260 (0.137)	0.267 (0.137)
Moderate	0.015 (0.142)	0.368 (0.138)**	0.213 (0.136)	0.392 (0.141)**	0.309 (0.141)*
Heavy	− 0.108 (0.166)	0.276 (0.161)	0.128 (0.158)	0.407 (0.163)*	0.214 (0.163)
*T* × light	0.065 (0.029)*	0.026 (0.030)	0.052 (0.021)*	0.123 (0.029)**	0.080 (0.028)**
*T* × moderate	0.066 (0.029)*	0.033 (0.030)	0.067 (0.021)**	0.146 (0.029)**	0.103 (0.028)**
*T* × heavy	0.071 (0.033)*	0.034 (0.034)	0.056 (0.023)*	0.120 (0.032)**	0.100 (0.032)**
*M*					
*T*	− 0.061 (0.015)**	− 0.080 (0.013)**	− 0.043 (0.011)**	− 0.142 (0.016)**	− 0.116 (0.014)**
Light	− 0.113 (0.102)	0.100 (0.098)	0.109 (0.098)	0.119 (0.096)	0.051 (0.097)
Moderate	− 0.012 (0.094)	0.037 (0.090)	0.094 (0.089)	0.170 (0.088)	0.091 (0.088)
Heavy	− 0.044 (0.099)	0.061 (0.094)	0.122 (0.094)	0.236 (0.092)*	0.134 (0.093)
*T* × light	0.003 (0.018)	− 0.002 (0.015)	0.004 (0.013)	− 0.003 (0.020)	0.009 (0.017)
*T* × moderate	0.012 (0.016)	0.018 (0.014)	0.013 (0.012)	0.038 (0.018)*	0.032 (0.016)*
*T* × heavy	0.029 (0.017)	0.026 (0.014)	0.018 (0.012)	0.044 (0.018)*	0.042 (0.016)**

Linear mixed-effects models adjusted for age, education, medical comorbidity and whether or not the administration of the cognitive tests was the first time ever; values presented are estimate (standard error)

*F*, female; *M*, male; *T*, time; Light/moderate/heavy, light/moderate/heavy intensity activity; ×, indicates interaction

*p < 0.05; **p < 0.01

## Discussion

In this population-based longitudinal study, we observed that participants engaging in non-exercise physical activity, irrespective of timing and intensity level, had significantly less decline than non-active participants across multiple cognitive domains. Of note, engaging in heavy intensity activity in either mid- or late-life as compared to being inactive was significantly associated with less decline of scores in all cognitive domains and engaging in moderate intensity activity in midlife was also associated with significantly less decline in all cognitive domains.

To our knowledge, our study may be among the first to show that engaging even in non-exercise physical activity such as household- and work-related activity is associated with less decline in cognitive trajectories across multiple cognitive domains in a large sample of persons aged ≥ 70 years from the community. Our results are partly in line with a recently published study from the UK that reported a beneficial effect of physical activity on cognitive trajectories of memory and executive function (Hamer et al. 2018). We also found that non-exercise physical activity, particularly when carried out at heavy intensity in either mid- or late-life, was associated with less decline in memory and attention. However, whereas the investigators from the UK reported that the association between physical activity and cognitive trajectories was more pronounced in women, we did not observe a consistent pattern in our data. When we stratified the analyses by sex, there were slightly more significant time x activity interactions for males as compared to females for midlife activity, but more significant time x activity interactions for females than males with regard to late-life activity. However, estimates might not have reached level of significance due to a low number of females reporting heavy intensity activity in mid- and late-life, as well as a low number of males reporting light intensity activity. The difference between the study from the UK and ours may be due to a different age range of participants (they included participants aged ≥ 50 years), different assessments used to determine cognitive function, and different follow-up time (their follow-up time was between 8 and 10 years and thus longer than ours). Furthermore, the questionnaire used in the other study assessed both exercise and non-exercise physical activity combined and regardless of timing, whereas we deliberately focused on non-exercise physical activity carried out at two different times in life (i.e., midlife and late-life).

In addition, researchers from the Northern Manhattan Study reported that cognitively unimpaired, ethnically diverse participants who engaged in low levels of leisure-time physical activity had a more pronounced decline in processing speed and episodic memory after 5 years of follow-up than participants who engaged in higher levels of leisure-time physical activity (Willey et al. 2016). However, their questionnaire only inquired about physical activity within two weeks of the first neuropsychological assessment. Therefore, our research adds to this study by showing that timing of physical activity may be an important factor to consider when investigating the association between physical activity and cognitive trajectories. Furthermore, a recent study among more than 6000 older adults found that higher levels of objectively measured moderate-vigorous intensity physical activity were associated with better maintenance in executive function and memory during an average of 3 years of follow-up (Zhu et al. 2017). It must be noted though that other studies have failed to establish an association between physical activity and change in cognitive function. For example, researchers from Rush University in Chicago studied a large sample of 4055 community-dwelling older adults and reported that while physical activity was associated with slower rate of cognitive decline after an average of 6 years of follow-up, this association was no longer significant after adjusting for cognitive activity and other covariates (Sturman et al. 2005).

Our study expands on the previous literature by showing that, on average, there was a higher number of significant time x activity interactions among *APOE* ε4 non-carriers indicating less decline on cognitive trajectories. This finding may be explained by a smaller sample size of *APOE* ε4 carriers that may have limited the power of our statistical analysis. However, it is also possible that, via yet unknown mechanisms, a potential beneficial effect of physical activity on cognitive function may be limited among *APOE* ε4 carriers and/or may be more pronounced in *APOE* ε4 non-carriers. Indeed, research has shown that a low level of physical activity is associated with a higher risk of cognitive decline, particularly among *APOE* ε4 carriers (Schuit et al. 2001). On the other hand, researchers from the University of North Carolina recently reported an improvement in memory function after an 8-month physical activity intervention irrespective of *APOE* ɛ4 carrier status (Etnier et al. 2018). However, interestingly, we observed in our data that engaging in midlife activity regardless of intensity level was associated with significantly less decrease in memory function only among *APOE* ε4 carriers.

The potential impact of sex on the association between physical activity and cognition has received growing interest in the field. Indeed, the National Institutes of Health (NIH) and other major health institutions have called for considering sex as a biological variable in research studies (Lee 2018). A recent review from Canadian researchers concluded that sex differences exist in dementia and associated risk factors such as genetics, cardiovascular factors, hormones or social and psychological factors. The researchers postulate that sex may also have an impact on the association between physical activity and cognition through mechanisms that are not yet clarified but may be related to neuroplasticity or neurotrophic factors (Barha and Liu-Ambrose 2018).

### Strengths and limitations

The major strength of our study is the longitudinal design that enabled us to assess cognitive trajectories over time. In addition, we evaluated a large sample of more than 2000 community-dwelling older persons. One limitation pertains to the self-reported questionnaire to inquire about non-exercise physical activity that may be prone to recall bias and/or social desirability bias. However, we derived the questions from validated instruments (the 1985 National Health Interview Survey, and the Minnesota Heart Survey intensity codes) (Folsom et al. 1985; Moss and Parsons 1986) and used a questionnaire that allowed us to assess not only late-life but also midlife non-exercise physical activity. Additionally, engagement in physical exercise may be a potential confounder. However, we did not adjust our analyses for physical exercise since we deliberately wanted to focus on non-exercise physical activity, and the correlations between physical exercise and non-exercise physical activity in our sample are only moderate. Furthermore, as in any observational study, we cannot establish a cause-effect relationship, and reverse causality may account for the observed association between non-exercise physical activity and less decline in cognitive trajectories. Thus, persons with incipient cognitive impairment may be less likely to engage in non-exercise physical activity, particularly in late-life, as compared to individuals free of cognitive impairment. Finally, we did not investigate potential mechanisms that may underlie the association between physical activity and cognitive trajectories in older adults. It has been discussed that the beneficial effects of physical activity on cognitive function may be due to various factors including but not limited to biomarkers such as increased release of growth factors, physiological factors such as increased cerebral blood flow and preserved cerebrovascular reserve, or psychological factors such as improved mood or sleep (Tyndall et al. 2018).

## Conclusion

We observed evidence of an association between non-exercise physical activity in midlife or late-life and less decline in cognitive trajectories. This association varied depending on the timing of engaging in non-exercise physical activity and the intensity of activity. Overall, the data point toward a stronger association between non-exercise physical activity carried out in midlife and at higher intensity levels with less longitudinal decline in cognitive performance. Furthermore, the association between non-exercise physical activity, particularly when carried out in late-life, and less cognitive decline appears to be more pronounced among *APOE* ε4 non-carriers. More research is needed to confirm these preliminary findings and to further investigate the potentially beneficial effect of non-exercise physical activity on cognitive trajectories in older adults.
